# Distribution and genetic characterization of hantaviruses in bats and rodents from Yunnan

**DOI:** 10.1371/journal.pntd.0012437

**Published:** 2024-08-29

**Authors:** Yutong Hou, Qian Li, Xingyu Huang, Jiale Wang, Junjie Hou, Yunze Sun, Xinrui Wu, Ziqin Dian, Binghui Wang, Xueshan Xia

**Affiliations:** 1 Faculty of Life Science and Technology, Kunming University of Science and Technology, Kunming, Yunnan, P.R. China; 2 Dali University, Dali, P.R. China; 3 Department of Clinical Laboratory, The First People’s Hospital of Yunnan Province, Yunnan, P.R. China; 4 School of Public Health, Kunming Medical University, Kunming, China; Chengde Medical University, CHINA

## Abstract

Hemorrhagic fever with renal syndrome caused by hantaviruses has long been a serious public health issue in Yunnan Province. Hantaviruses exhibit a high extent of biodiversity in their natural hosts, particularly in mammalian hosts. This study was conducted to screen for hantaviruses in bats and rodents in Yunnan Province and elucidate their genetic characteristics and possible zoonotic disease risk. Hantaviruses were detected in 202 bats and 372 rodents with the positive rates 27.49% and 1.25% respectively. A novel lineage (named Lineage 10) of the Seoul virus (SEOV) from rodents and the geographic clustering of hantavirus in bats were identified using phylogenetic analyses of the full-length M- and S-segments. Our study suggest a high cross-species transmissibility of hantaviruses in bats and existence of a new lineage of SEOV in rodents differing significantly from other SEOVs. These results provide data to support the prevention and control of hantavirus-associated diseases in Yunnan Province.

## Author summary

Hantavirus is an important pathogen that can cause hemorrhagic fever with renal syndrome (HFRS) and hantavirus cardiopulmonary syndrome (HCPS). HFRS has long been a serious public health problem in Yunnan Province. However, the molecular epidemiology and host characteristics of hantavirus in Yunnan Province are still unclear. Therefore, we screened hantaviruses in bats and rodents in selected areas of Yunnan from 2018 to 2022 and found the 10th SEOV lineage in rodents, as well as new geographic clusters of Xuan son virus (XSV) and novel hantavirus in bats. We proposed the possibility of the 10th SEOV lineage in rodents as an ancestral lineage and explained the related host-switching events in bat hantaviruses. These results provide data to reveal a greater risk of zoonotic disease and support the prevention and control of hantavirus-associated diseases and in Yunnan Province.

## Introduction

Hantaviruses are enveloped, segmented, negative-strand RNA viruses of the *Hantaviridae* family. Its virions are generally spherical in nature, with an average diameter of approximately 80–120 nm. Hantavirus genomes consist of three segments, designated L (large), M (medium), and S (small). The L-segment encodes the RNA-dependent RNA polymerase (RdRp), the M-segment encodes a glycoprotein precursor that is further processed to produce two transmembrane glycoproteins, Gn and Gc, and the S-segment encodes the nucleocapsid protein [1].

The first isolation of hantaviruses was reported from the *Apodemus agrarius* near the Hantan river in Korea [2]. The natural mammalian hosts of hantaviruses include Chiroptera, Murinae, Arvicolinae, Soricomorpha, and Insectivora [3]. Fifty types of hantaviruses have been identified worldwide, and at least 28 cause human disease [4]. To date, only rodent-borne hantaviruses are responsible for the human diseases hemorrhagic fever with renal syndrome (HFRS) and hantavirus cardiopulmonary syndrome (HCPS) [5]. The main clinical symptoms of HFRS are hemorrhage, petechiae, inflammatory symptoms of the eye, acute myopia, and varying degrees of acute renal failure. The clinical symptoms of HCPS include dry cough, rapidly worsening dyspnea on chest radiography, and rapidly evolving bilateral interstitial edema [6].

The current mainstream classification of hantaviruses is based on geographical distribution, namely "old world" and "new world" types. The old world hantaviruses are mainly prevalent in Europe and Asia and are carried by rodents and insectivores, resulting in HFRS [4]. The most widespread hantavirus in Europe is Puumala virus (PUUV) carried by *Myodes glareolus* [7], and most cases of HFRS associated with PUUV have been diagnosed in Europe, Russia, Finland, Sweden, France, Germany, and the Balkans [8]. The outbreak caused by PUUV reached in a new incidence peak in Germany in 2012 with more than 2,800 reported cases [9]. Dobrava-Belgrade virus (DOBV) infection occurs mainly in southeastern Europe and has been the cause of almost all cases of HFRS in Greece. DOBV is the most fatal hantavirus in Europe, with a 12% case fatality rate [10]. Other rodent-borne hantaviruses that cause severe HFRS, Hantaan virus (HTNV), and the HTNV-like Amur/Soochong virus, are transmitted in Far East Russia, China, and South Korea [11]. The Seoul virus (SEOV), harbored by *Rattus norvegicus*, is unique among old world hantaviruses in that it spreads worldwide because of the highly migratory and global distribution of its host [12]. The new world hantaviruses, predominantly prevalent in the Americas, cause HCPS. The first isolate was identified in 1993 after an outbreak of acute pulmonary distress syndrome in the United States, from *Peromyscus maniculatus*, and was named Sin Nombre virus [13]. Different hantaviruses have been identified and isolated in the Americas [14], with the predominant one in South America being the Andes virus (ANDV), which caused an epidemic of HCPS in Argentina [15]. More than 30 hantaviruses have been identified throughout the Americas [5]. The same hemorrhagic fever is commonly caused by multiple different genotypes of hantaviruses. In Brazil, the hantaviruses that cause HCPS are complex, including at least seven genotypes, the most virulent of which is Araraquara virus, with a 50% mortality rate [16]. Therefore, the discovery of novel hantaviruses and their different genotypes is highly significant for clinical research.

The majority of hantavirus hosts are distributed in China, and SEOV and HTNV, carried by rodents, have been prevalent in recent years [17]. SEOV is widely distributed worldwide and divided into nine lineages, with lineages 1, 3, and 5 being mainly from China [18]. Multiple bat-borne hantaviruses have also been found in Guangxi, Yunnan, Hubei, and Zhejiang in China, indicating a potential disease risk [3,19,20].

Yunnan Province is a geographically diverse region located at the crossroads of several countries, its environment provides sufficient conditions for virus hosts to survive and facilitate the spread of related diseases which is supported by studies of Yunnan bats harboring coronaviruses with genomic similarity to the novel coronavirus [21] and by reports of perennial epidemics of the plague in Yunnan [22]. Therefore, this study was conducted to screen for hantaviruses in bats and rodents in Yunnan Province and elucidate their genetic characteristics and possible transmission risk through a gene sequence analysis of epidemic strains. The overall aim was to provide scientific data to support infectious disease prevention and control in Yunnan Province.

## Materials and methods

### Ethics statement

This study was approved by the Institutional Ethical Committee of Kunming University of Science and Technology (protocol no. 16048). Rare and protected animals were not involved.

### Sample collection

Bats and rodents were captured from wild environments or suburbs in various prefectures of Yunnan Province, including Kunming, Chuxiong, Honghe, Dehong, Nujiang, Xishuangbanna and Dali. They were transported with dry ice, and stored at -80°C for further laboratory processing. They were then dissected, and lung tissue was removed and placed in virus preservation solution and stored at -80°C until RNA extraction. Bat and rodent sampling locations are listed in S1 Table.

### Hantavirus screening

Tissue suspension were prepared through grinding and centrifugation. Total RNA was extracted from 200 μL of homogenates using a TIANamp virus RNA kit (TIANGEN). Universal primers [23] were used to amplify the hantavirus RdRp gene (partial L-segment), which was sequenced for further phylogenetic analysis at Tsingke Biological Co. Ltd.

### Amplification and sequencing of full-length M- and S-segments of hantavirus

Following the initial identification of the RdRp gene using BLAST (NCBI), primers were designed to amplify the full-length hantavirus M- and S-segments in rodents and bats based on conserved regions. S2 Table presents all the primers used to amplify the M- and S-segments. The partial L-segment and full-length M-, and S-segments sequences for hantavirus obtained in this research were submitted to NCBI GenBank. S3 Table provides accession numbers of published sequences for all strains.

### Phylogenetic analyses

ClustalW in MEGA-X was used to align multiple sequences, and phylogenetic analysis was performed using maximum likelihood (ML) trees (GTR + G + I and 1000 bootstrap) in MEGA-X for the partial L-segment of all positive samples and the full-length M- and S- segments of the new subtypes and novel hantaviruses. Dobrava-Belgrade virus strain Afl9/1999 and PUUV strain DTK/Ufa-97 were used as the outgroup for new subtypes of SEOV and bat-borne hantaviruses, respectively. Estimates of genetic divergence were obtained via pairwise analysis using MEGA-X for the full-length M- and S-segments of hantavirus variants. In addition, an amino acid phylogenetic analysis (Poisson model and 1000 bootstrap) of bat hantaviruses was constructed to complement the above arguments in S1 Fig.

## Results

### Hantavirus infections in rodents and bats

Between 2018 and 2022, a total of 372 rodents were collected from eight prefectures in Yunnan Province, as detailed in S1 Table. The sample included 157 *Rattus norvegicus*, constituting 42.20% of the total, 170 *Rattus tanezumi* (45.70%), and twenty *Apodemus ilex* (5.38%). Additionally, 202 bat lung samples were obtained, with 123 samples (60.89%) from *Hipposideros gentilis*, twenty (9.90%) from *Hipposideros armiger*, nineteen (9.40%) from *Rhinolophus sinicus*, and thirteen (6.4%) from *Rhinolophus affinis*, collected across five prefectures in Yunnan Province. Hantaviruses were identified in bats and rodents, with positive rates of 27.49% (47/202) and 1.6% (6/372), respectively. The positive rates in bats were 31.7% (45/142) in Nujiang and 6.90% (2/29) in Pu’er and the positive rate was 25% (5/20) in *R*. *norvegicus* in Kunming and 5% (1/20) in *R*. *tanezumi* in Honghe.

### Genetic characteristics of Hantavirus strains

**Fig 1:**
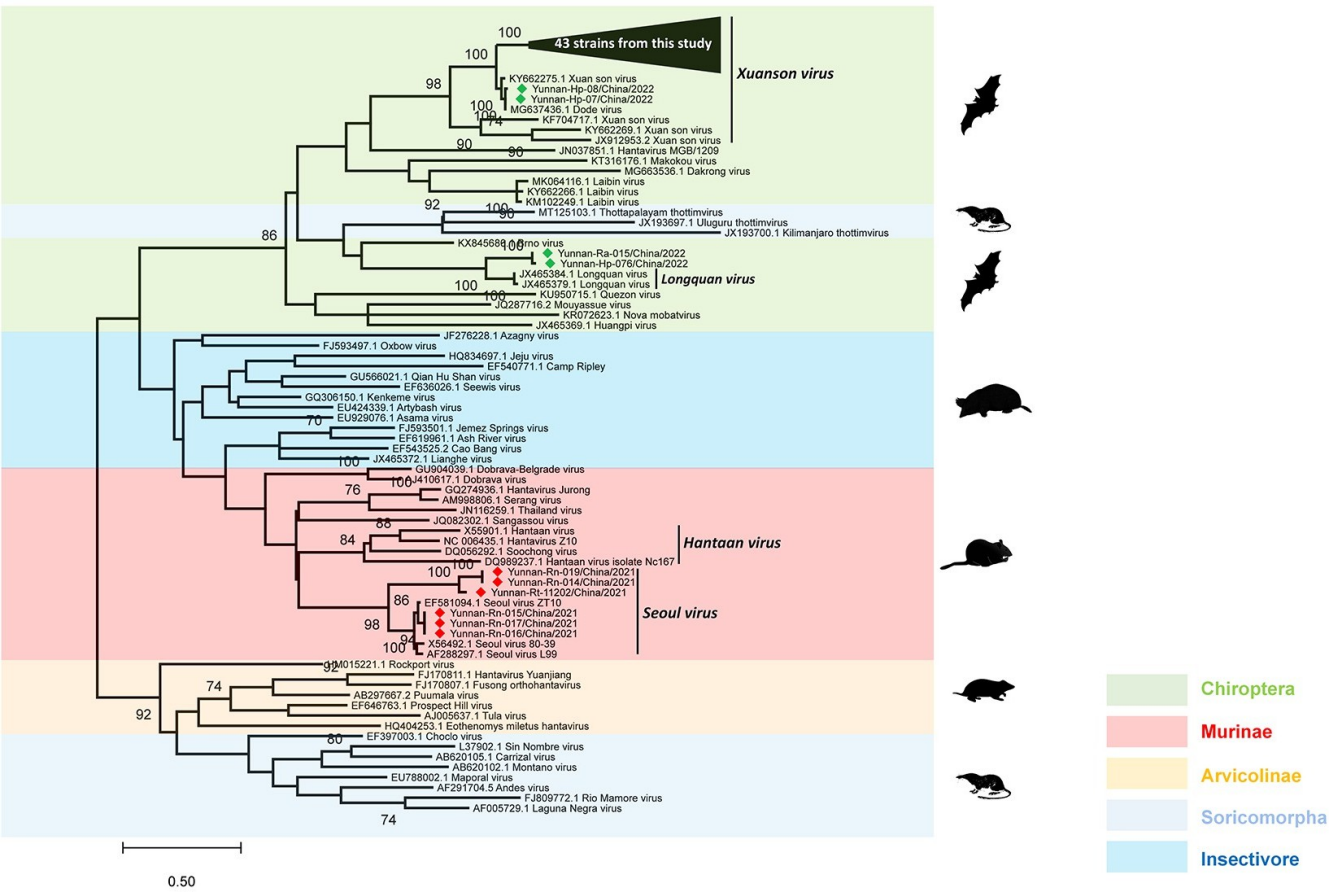
Maximum-likelihood phylogenetic tree based on partial L-segments of Mammalian hantavirus. The tree was constructed using hantavirus **L-segments** showed all strains divided into 5 phylogroups: Chiroptera, Murinae, Arvicolinae, Soricomorpha, Insectivore, with the Soricomorpha and Arvicolinae in a relatively basal position.

Hantaviruses are primarily hosted by mammals, with pathogenic strains predominantly associated with Muridae. For preliminary hantavirus identification, a maximum likelihood phylogenetic tree based on L gene sequences was constructed using five phylogroups of hantaviruses carried by different mammalian hosts: Chiroptera, Murinae, Arvicolinae, Soricomorpha, and Insectivora, with Soricomorpha and Arvicolinae positioned relatively basally (Fig 1). The results indicate that hantaviruses associated with Murinae and Insectivora exhibit close phylogenetic relationships. Chiroptera forms a distinct cluster; however, Thottapalayam thottimvirus (TPMV), Uluguru thottimvirus (ULUV), and Kilimanjaro thottimvirus (KMJV), harbored by Insectivora, act as a separate cluster within the Chiroptera phylogroup. Soricomorpha and Arvicolinae cluster together and are positioned basally in the phylogenetic tree. Upon adding the L gene sequences obtained in this study, forty-five strains from bats were XSV, and other two strains were novel hantaviruses. Of the 45 XSV strains from bats, 43 were identified in *Hipposideros gentilis*, one in *Rhinolophus sinicus*, and one in *Hipposideros armiger*. All strains from rodents were SEOV.

The two bat-borne hantavirus sequences from Pu’er formed a region-specific phylogroup with the XSV strain Dode virus in Pu’er, and the forty-three bat-borne hantavirus sequences from Nujiang formed a single branch with the closest affinity to this phylogroup. In addition, the other two sequences from Nujiang, respectively identified in *Rhinolophus affinis* and *Hipposideros gentilis*, were in separate clusters and were distantly related to other sequences found in Nujiang, with the closest related branch being the Longquan virus (LQUV) found in Zhejiang [3]. The rodent-borne hantaviruses in this study were SEOV, and the three sequences from Kunming clustered with Seoul lineage 3 found in Zhejiang, whereas the two sequences from Kunming and one from Honghe clustered to form a branch, and all were relatively distant from the known SEOV (Fig 1). Only the new geographic clusters and novel hantavirus were subjected to subsequent full-length genomic analysis.

### Identifications of a novel rodent-borne Seoul Virus lineage

**Fig 2:**
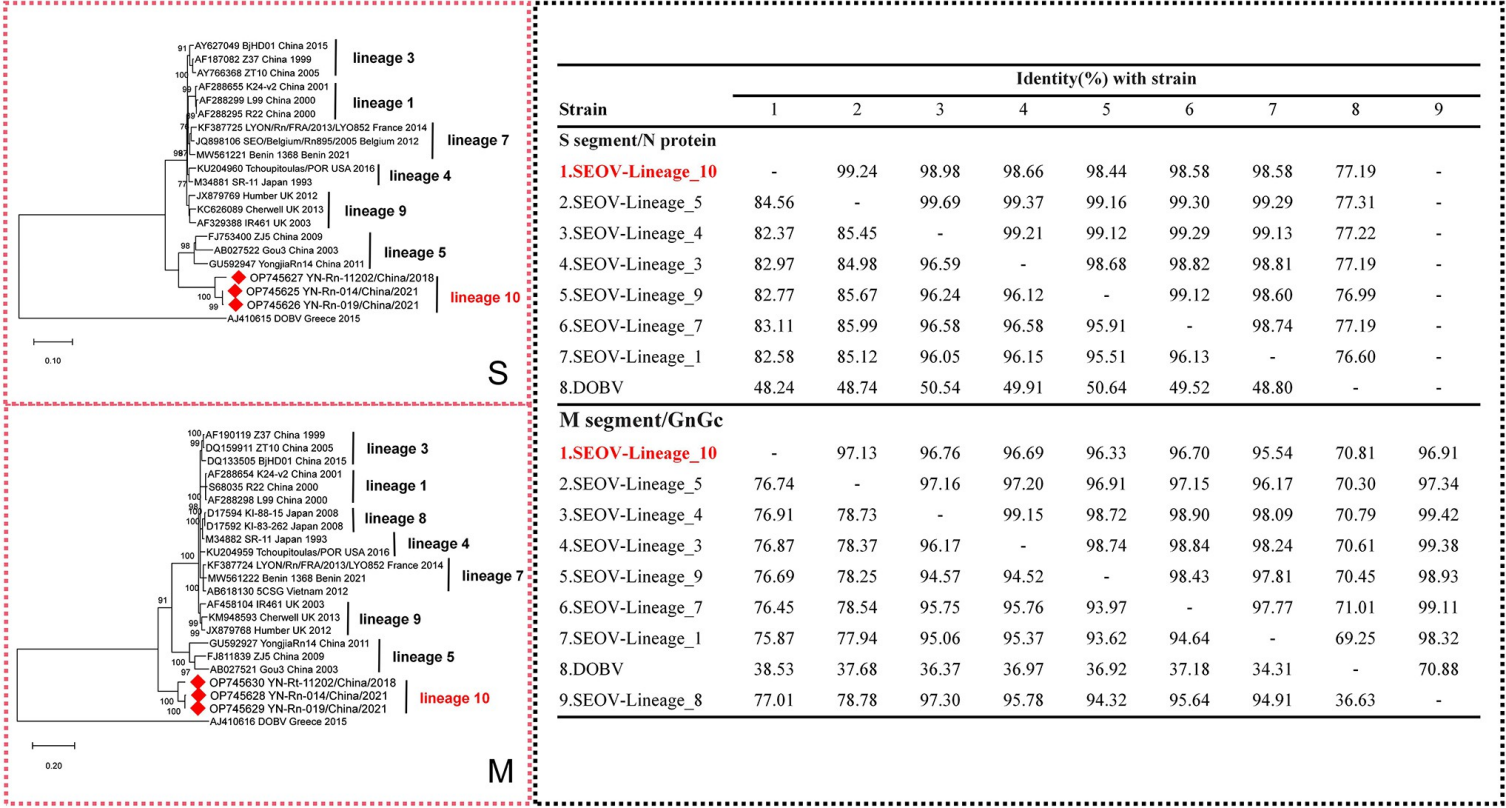
**Maximum-likelihood phylogenetic tree based on full-length M- (A) and S-segments of Seoul virus (B).** This tree was rooted using DOBV as an outgroup and the **full-length M-** and **S-segments** of SEOV strains identified fell into 7 and 8 lineages, respectively. Only bootstrap support of ≥70% are shown. **Comparison of Encoded Amino Acid Sequences of the M- and S-segments of Seoul virus(C)**.

A phylogenetic analysis of rodent-borne hantavirus sequences was performed based on full-length M- and S-segments, including three strains belonging to new lineages in the RdRp gene phylogenetic analysis. Herein, M- and S-segments of SEOV strains identified fell into seven and eight lineages, respectively (missing lineages did not provide complete genome, and lineage 8 did not have full-length M-segments). In the phylogenetic ML tree based on full-length M- and S-segments of SEOVs, the three SEOV strains identified herein formed a new lineage (ML bootstrap showed high support) and shared a genetically distinct position with lineage 5 found in Zhejiang (Fig 2A) [24]. The ML tree based on the full-length M-segments produced a similar topology to that based on the S-segments, and the new lineage clustered individually at a most basal position compared with other SEOVs. These findings suggest that the M-segments of the new lineages exhibits a higher degree of variation compared to the S-segments. (Fig 2B).

The nucleotide and amino acid sequence similarities of the entire S (1,290 nt) and M (3,402 nt) CDSs of the novel lineage with other lineages were between 82.37/98.44–84.56/99.24% and 75.87/95.54–77.01/97.13%, respectively (Fig 2C). The range was smaller than the range of nucleotide and amino acid differences, confirming that the SEOV identified herein is the 10^th^ lineage, with a close evolutionary relationship with Gou virus (GOUV) found in Zhejiang Province. And these two lineages differ significantly from other lineages of SEOV, indicating the existence of a genetically distinct SEOV phylogroup.

### Phylogenetic analysis of full-length M- and S-segments of bat-borne Hantavirus

**Fig 3:**
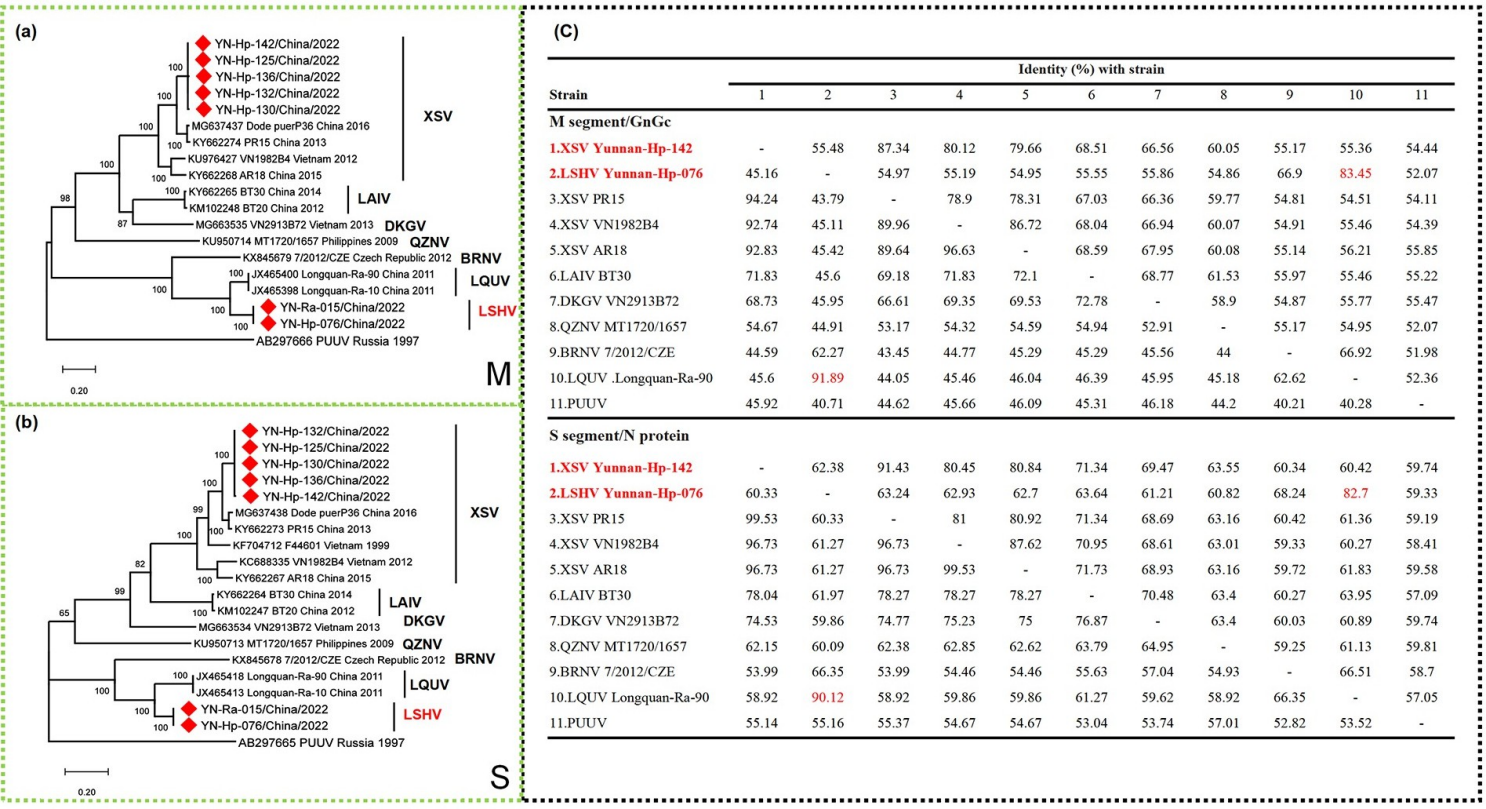
**ML tree based on full-length M-segments of bat-borne hantavirus (A). ML tree based on full-length S-segments of bat-borne hantavirus (B).** This tree consists of all the bat-borne hantavirust hat have been reported to have full-length M- and S-segments (XSV, LAIV, QZNV, DKGV, BRNV, and LQUV), with PUUV as the outgroup. Only bootstrap support of ≥70% are shown. Comparison of Encoded Amino Acid Sequences of the full-length M- and S-segments of bat-borne hantaviruses(C).

Given the high similarity among the 43 XSVs, only 5 were chosen along with the 2 strains related to LQUVs for M- and S-segments amplification and construction of phylogenetic ML trees with the full-length M- and S-segments of bat-borne hantaviruses The ML trees produced similar topologies; (Fig 3A and 3B). The XSV found in Nujiang in this study was closely related to, but did not cluster with, the PR15 strain found in Pu’er, Yunnan. Herein, the Dode virus was also from Pu’er, Yunnan, but only approximate full-length segments were provided. To verify that XSV forms two distinct geographic clusters in Yunnan, the Dode virus was included as a reference.

LQUV and Brno virus (BRNV) do not belong to Mobatvirus in the genus-level classification, but are separately classified as Loanvirus, and the phylogenetic analysis was consistent with this classification. The two strains showed a close phylogenetic relationship with LQUV, sharing relatively basic phylogroups (ML trees based on M- and S- segments both produced very similar topologies). The ML tree constructed from the corresponding amino acid sequences supports the above conclusion. (S1 Fig).

Based on nucleotide and amino acid homology statistics for the sequences encoding G and N proteins, respectively, the new bat-borne hantaviruses exhibited 91.89% and 90.12% amino acid similarity to the full-length M and S genes of the most related strain, LQUV Longquan-Ra-90 (Fig 3C). This is consistent with the International Committee on Taxonomy of Viruses (ICTV) requirement for hantavirus species demarcation, which states that a new virus must differ by more than 7% from other hantaviruses based on N and G protein amino acid sequences [25]. Therefore, we named this new virus Lushui virus (LSHV).

Among these, the new geographic cluster YN-HP-142 of XSV (the remaining four strains encoded the same protein) has an entire S-segment CDS region (Nucleocapsid protein) of 1,284 nt and an entire M-segment CDS region (Glycoproteins) of 3,387 nt. LSHV, a new member of the genus Loanvirus, has an entire S-segment CDS region of 1,272 nt and an entire M-segment CDS region of 3,402 nt. LSHV is consistent with other members of the genus Loanvirus. Specifically, in the S gene, the Loanvirus genus lacks 3 amino acids at positions 226–234 nt and 1 amino acid at positions 754–757 nt, compared to other bat-borne hantaviruses. In contrast, the GP gene of the Loanvirus genus exhibits an additional amino acid at positions 967–969 nt.

## Discussion

Hantaviruses, members of the Hantaviridae family within the order Bunyavirales, represent a large and diverse group with a wide host distribution in nature. Significant genetic variations exist among virus strains from different animal hosts. Notably, rodent-borne hantaviruses, such as those belonging to the Hantaan and Seoul virus groups, are capable of infecting humans and causing diseases. The spectrum of illnesses caused by hantaviruses varies depending on the specific virus involved. For example, ANDV causes severe HCPS; however, Prospect Hill virus is not associated with human disease [26].

In China, a high prevalence of hemorrhagic fever with renal syndrome (HFRS), primarily caused by SEOV and HTNV, has been reported, leading to major epidemics. Notably, Yunnan Province in China has consistently been an endemic region for hantavirus infections [27]. In 2020, areas with high HFRS prevalence were predominantly Dali and Chuxiong states, accounting for 89.20% (190/213) of the cases in Yunnan. From 2005 to 2012, the population prevalence of HFRS was higher in Kunming (13.95%, 30/215) and lower in Honghe (8.37%, 18/215) [28]. The positivity rates of SEOV identified in this study are consistent with these findings: 25% for *R*. *norvegicus* in Kunming and 5% for *R*. *tanezumi* in Honghe.

The epidemic hantaviruses reported in China are mainly SEOVs harbored by *R*. *norvegicus* and HTNV harbored by *Apodemus agrarius* [24], but the SEOV identified in this study is still the most widely distributed hantavirus globally, which has developed from a single epidemic in Asia to a four-continent epidemic in Asia, Europe, America, and Africa [29]. Bat-borne hantaviruses, such as Laibin virus found in Guangxi [30], Huangpi virus found in Wuhan, and LQUV found in Zhejiang [3], have been frequently found in China, indicating that China has become a priority area for the prevalence of bat-borne hantaviruses. In recent years, numerous novel hantavirus strains have been discovered and identified from various small mammal hosts in Yunnan [19,31–33]. Here, we screened hantaviruses in bats and rodents in selected areas of Yunnan from 2018 to 2022 and found the 10th SEOV lineage in rodents, as well as new geographic clusters of XSV and the novel hantavirus LSHV in bats, revealing a greater risk of zoonotic disease in Yunnan.

Hantaviruses were thought to be host-specific, with one hantavirus generally corresponding to only one rodent host [34]. Recently, several host-switching events have occurred for HTNV and SEOV [24]. The discovery of a homologous SEOV lineage 10 (with Yunnan-Rn-014 in Kunming and Yunnan-Rt-11202 in Honghe having M-CDS and S-CDS similarity of 98.24% and 99.3%, respectively) in both *R*. *tanezumi* and *R*. *norvegicus* also revealed a host spillover event, from *R*. *norvegicus* to *R*. *tanezumi*, helping to elucidate the evolutionary pattern of hantaviruses.

As mentioned, lineage 10 and 5 together form an underlying phylogroup and show early evolutionary divergence. It has been reported that the middle and lower reaches of the Yangtze River, where lineage 5 barbored by *R*. *norvegicus was* identified, were the radiation centers for most known phylogenetic lineages of SEOVs in China. Contrary to this conclusion, lineage 10 is clearly in a more basal position, especially in the phylogenetic analysis of full-length M-segments. Ruling out the occurrence of recombination events in the M-segment, we inferred that lineage 10 might be a considerably earlier ancestor of SEOV.

Bats are important potential hantavirus hosts, and their ability to fly long distances, population densities, and sociality favor the efficient maintenance, evolution, and spread of viruses [3]. XSV was first reported in 2012 in *H*. *gentilis* in Phu Tho Province, Vietnam [35], later in Pu’er in 2013 [19], and subsequently in *H*. *cineraceus* in Laibin, Guangxi in 2015 [20]. Herein, it was reported in Nujiang in 2022. But based on the partial L-segment, full-length M-, and S-CDS differentiation order, XSVs from Laibin and Vietnam are speculated to be basic and possibly the ancestors of XSVs. Based on the genetic differentiation sequence and timeline, XSV in Nujiang was likely transmitted by H. gentilis from Phu Tho. Among the 43 XSV-positive samples in Nujiang, 41 were from *H*. *gentilis*, while the remaining 2 were from *Rhinolophus sinicus* (Yunnan-Rs-117/China/2022) and *Hipposideros armiger* (Yunnan-Ha-033/China/2022). This indicates that a host-switching event may have occurred within the local bat population.(S1 Table). We speculate that efficient virus transmission in Nujiang might be associated with higher local population densities, and the three bat caves sampled were all in the Gaoligong Mountain National Nature Reserve of Lushui City and in close proximity to each other.

Of note, LSHV was found simultaneously with XSV in the Nujiang bat samples from this study, with a much smaller positive rate than XSV. Considering that its hosts include *R*. *affinis* (Yunnan-Ra-015/China/2022), there is strong evidence that LSHV has a different transmission route than XSV. Apart from LSHV, there are only two viruses belonging to Loanvirus worldwide, LQUV carried by *Rhinolophus* found in Zhejiang, China in 2011 [3], and Brno virus carried by *Nyctalus noctula* found in the Czech Republic in 2012 [36]. The ML tree results showed that LSHV, LQUV, and BRNV are in a relatively basal position; however, since the host of BRNV is *Nyctalus noctula*, it is currently unclear whether there is a transmission relationship between LSHV and LQUV in China and BRNV in the Czech Republic. (Fig 3A and 3B).

Yunnan, a province of China bordering Laos, Myanmar, and Vietnam, has a very important geographical location, and owing to its rich flora and fauna, the main hosts of hantaviruses interact frequently. Therefore, we must not consider only the invasion of hantaviruses carried by rodents, including SEOV and HTNV, which cause HFRS epidemics in China and East Asia, but also prevent and control the entry of hantavirus-carrying species from other countries. Recently, SEOVs originating from East Asia have been reported successively in Europe [18], but the migratory transmission pathways of their rodent hosts are not known. Yunnan is located in an intermediate geographical position; thus, the composition and prevalence of its rodent hosts must be further elucidated.

One of the limitations of this study is the lack of comprehensive rodent hantavirus-positive samples, preventing an overall assessment of the trend of hantavirus prevalence in Yunnan. Given the critical situation in Yunnan, which is an original outbreak area for HFRS and expanding yearly, we plan to collect more samples of potential hantavirus carriers and expand the screening scope to investigate the prevalence of pathogenic hantaviruses carried by rodents while monitoring the prevalence of hantaviruses in other hosts, especially bats capable of long-distance flights. Ultimately, these data will support the prevention and control of hantavirus-associated diseases in Yunnan Province.

## Supporting information

S1 Fig ML tree constructed from the amino acid sequences corresponding to the entire M and S CDSs of bat-borne hantaviruses. (TIF)

S1 Table Prevalence of hantavirus in bats and rodents by species and location in Yunnan. (XLSX)

S2 Table The primers for amplification of the full-length M- and S-segments. (XLSX)

S3 Table Accession numbers of published sequences for all strains. (XLSX)
