# Supplementary figures and images for: Distribution and genetic characterization of hantaviruses in bats and rodents from Yunnan
Source: PLoS Negl Trop Dis. 2024 Aug 29;18(8):e0012437. doi: 10.1371/journal.pntd.0012437 (PMC11412632; doi:10.1371/journal.pntd.0012437)

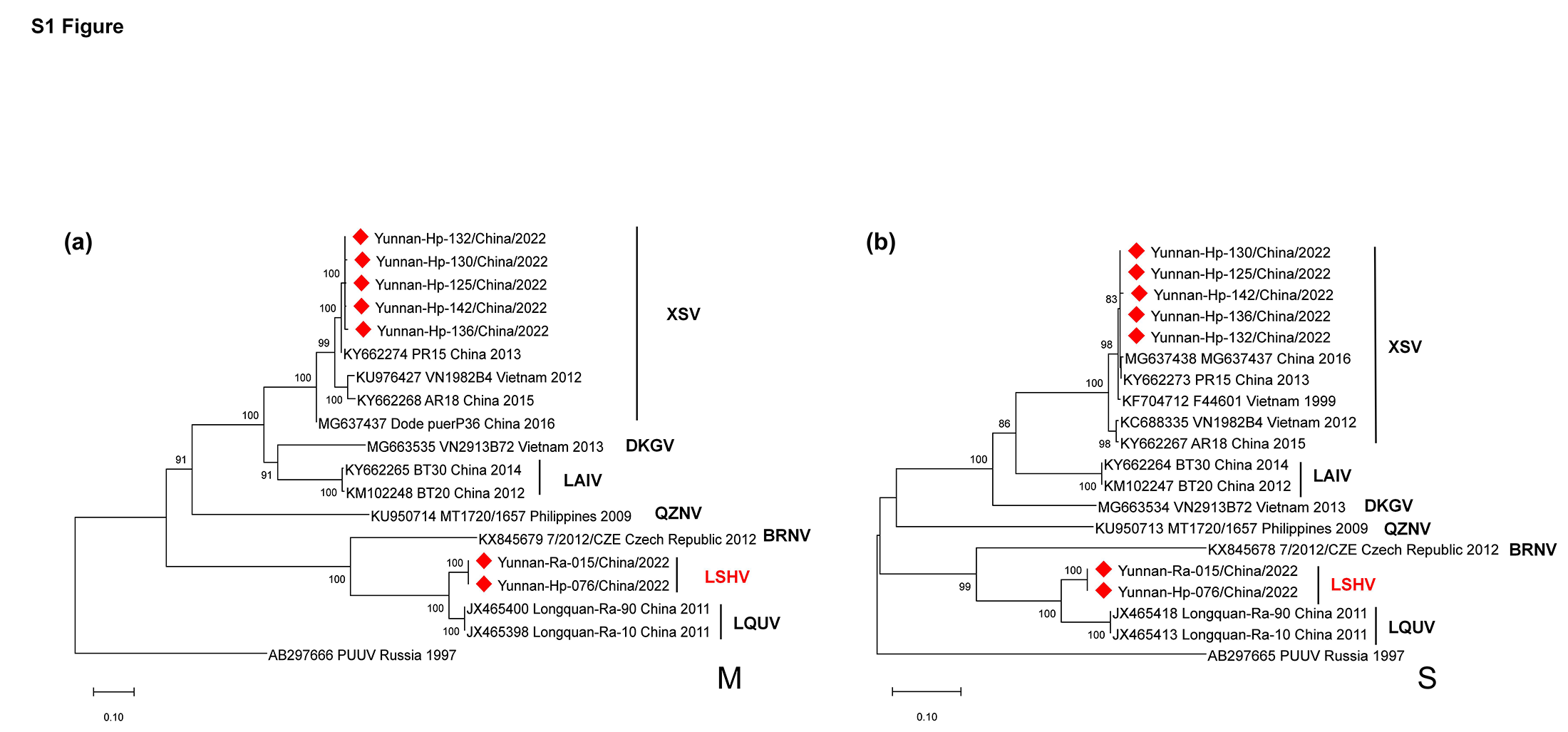

Supplement: S1 Fig — (TIF) [file pntd.0012437.s001.tif]
