# Supplementary material for: Distribution and genetic characterization of hantaviruses in bats and rodents from Yunnan
Source: PLoS Negl Trop Dis. 2024 Aug 29;18(8):e0012437. doi: 10.1371/journal.pntd.0012437 (PMC11412632; doi:10.1371/journal.pntd.0012437)
Supplement: Attachment — Submitted filename: resubmission_cover_letter-5.3.docx [file pntd.0012437.s005.docx]

2024.5.3

Wen-Ping Guo

Academic Editor

*PLoS Neglected Tropical Diseases*

Dear Editor:

We wish to re-submit the manuscript titled “Distribution and Genetic Characterization of Hantaviruses in Bats and Rodents from Yunnan.” The manuscript ID is PNTD-D-23-01645R1.

We thank you and the reviewers for your thoughtful suggestions and insights. The manuscript has benefited from these insightful suggestions. I look forward to working with you and the reviewers to move this manuscript closer to publication in *PLoS Neglected Tropical Diseases*.

The manuscript has been rechecked and the necessary changes have been made in accordance with the reviewers’ suggestions.the modifications are highlighted in yellow in the revised manuscript. The main corrections in the manuscript and the responds to the reviewer’s comments are as follows:

**Response to Reviewer 's Comments**

**Methods**

**Reviewer #1**: The authors have made phylogeographic claims. In such cases, I believe that geographical information such as map information is essential.

**Response:** We have added relevant geographic information in Sample Collection. (Lines 115-120)

Although this study determined the full-length sequences of the S and M segments, it did not present any primer information used for this determination. If NGS is used, the method should be described; however, this method has not yet been described.

**Response:**We have added S2 Table to provide all primers for amplification of the M and S segments. (Lines 131)

H. pomona, described by the authors, is now referred to by a different scientific name. Please check the reference and provide the correct species name. Diversity 2021, 13, 218. <https://doi.org/10.3390/d13050218>

**Response:** Regarding the name of *H. pomona*, we consulted the literature you provided and changed H. pomona to *H. gentilis* in the full text.

Reviewer #2: Objectives of the study are clearly articulated, study design is appropriate to address the stated objectives. Methods are clearly described and appropriate, additional clarify is needed regarding sampling. Sample size is adequate for this type of study. Phylogenetic methods are correct Specific commenst:

Line 44 - please, refer to the current hantavirus taxonomy;

**Response:**We reclassify hantaviruses in the Hantaviridae family of the Bunyaviralese. (Lines 52-53)

Line 50 - the statement "Hantaviruses originated from the black threaded gopher" seems incorect, please, revise and clarify ;

**Response: W**e've changed it to "The first isolation of hantaviruses was reported from the Apodemus agrarius near the Hantan river in Korea". (Lines 59-60)

Line 68 - the statement "The last PUUV outbreak occurred in Germany in 2012" is not true, please, update the references and revise;

**Response:** We've changed it to “The outbreak caused by PUUV was reached in a new incidence peak in Germany in 2012 with more than 2,800 reported cases”. (Lines 75-77)

Line 69 - Dobrava virus (DOBV) - please, correct the full name to Dobrava-Belgrade virus

**Response:** We've corrected the full name to Dobrava-Belgrade virus in the full text.

Line2 102,103 - the statement is unreferenced, please, clarify

**Response: S**ince that statement is not referenced, we decided not to describe lineage 10 in Introduction.

In the Results sections analyses of fecal samples are presented yet no mention of this type of samples in the methods

**Response:** We initially screened for hantavirus in multiple tissues of a sample including lung, intestinal, and fecal samples, where lung samples had the highest positivity rate, and therefore we revised the source of bat samples to lung tissue in the full text. (Lines 118,149).

**Results**

Reviewer #1: The resolution of the figure and tables was poor and indiscernible (figures 1-3). Some action is needed.

**Response:** We modified the resolution to make the image clearer following the guidelines.

The classification of LQUV and BRNV is mentioned, but it should be discussed using amino acid-based phylogenetic analysis, as well as DNA phylogenetic analysis. The authors may be correct in their assertion, but it is not clear whether they are misrepresenting the facts, and it is impossible to discern. This should be mentioned based on the phylogenetic analysis using amino acids.

**Response:** Figure 3 also provides data on amino acid homology, but given the necessity of amino acid developmental analysis as you mentioned, we decided to construct an amino acid tree in Supplementary Figure 1. (Lines 142-144)

In the phylogenetic analysis of hantaviruses in figure 1, there are several viruses that should be included as outgroups. Among them, Thottapalayam thottimvirus (TPMV), Nova mobatvirus (NVAV), Uluguru thottimvirus (ULUV), and Kilimanjaro thottimvirus (KMJV) must be included, and in this study, thottimvirus group was not added at all. In particular, ULUV and KMJV are the only viruses identified in Myosoricinae, so author must be included in the phylogenetic analysis of hantaviruses as a whole, but they were not included in this study.

**Response:** We have added the Thottapalayam thottimvirus (TPMV), Nova mobatvirus (NVAV), Uluguru thottimvirus (ULUV), and Kilimanjaro thottimvirus (KMJV) you suggested to the ML tree in Fig 1.(Lines 160-162)

In a normal phylogenetic analysis, multiple branches usually indicate inadequate analysis. There is a region of low bootslap (no numbers) and multifurcation downstream of bootstrap 86 in the authors' figure 1, Chiroptera region. Analysis of this portion of the region is entirely uncertain, and it is impossible to draw definitive conclusions from these results. Figure 1 also confirms the results of the low bootstrap values (no numbers) in areas where hantaviruses detected from each organism are heavily branched. This result alone could lead one to mistakenly believe that the large categories shown in figure 1 are not sufficiently reliable. Reanalysis of the authors' data with additional sequence data might provide results that support the authors' assertions.

**Response:** The problem of low bootstrap values for the individual branches in Fig. 1 is indeed natural consequence, and the inclusion of additional sequences could lead to even lower bootstrap values, therefore, here we presented the original tree without hiding the bootstrap values below 70% and .phylogenetic analysis from other article which has the similar topologies regarding to Chiroptera region to prove the reliability for Fig 1.

**Fig 1. Maximum-likelihood phylogenetic tree based on partial L segments of Mammalian hantavirus without the hiding of bootstrap values.**

Fig 3 in ***Molecular Phylogeny of Mobatviruses (Hantaviridae)in Myanmar and Vietnam***: Phylogenetic trees, based sequences of the S-, M-, and L-genomic segments, respectively, generated by the Bayesian Markov chain Monte Carlo estimation method, under the GTR + I + Γ model of evolution

**Specific comments:**

In the Method s section preparation of lung samples is described, yet Results are shown for fecal samples only? Please, apend and clarify

**Response:** We standardized our sample sources, using lung tissue samples from bats and rats in this study.(Lines 115-120)

Two headings in the results section are consecutively numbered 3.3, please, correct

**Response:** We've corrected this point.

Lines 214, 215 "five strains from the 42 viruses in separate clusters and two strains with closer affinity to 215 LQUV in Nujiang were selected" - selected for what? **please, append and clarify**

**Response:** .We explained that based on the phylogenetic analysis of the RDRP gene, 42 XSVs and two LQUVs were identified. Given the high similarity among the 42 XSVs, only five were chosen along with the two LQUVs for full-length amplification. (Lines 196-199).

**Conclusions**

Reviewer #1: Overall, the results were very interesting, but the phylogenetic and phylogeographic analyses were insufficient to fully understand the path to the conclusions. I request that the authors conduct the analysis again.

Reviewer #2: Conclusions are missing and are not clearly formulated

Response: We will reconsider the accuracy of the conclusions and have amended the relevant statements.For example, we reorganize our conclusions about lineage 10 as the earlier ancestor of SEOV and remove the statement that the Bruno virus is far from China. (Lines 272-277,320-322).

**Editorial and Data Presentation Modifications?**

Reviewer #1: As noted in the Methods section, primer information is not provided, but information on the deposited sequences is provided in supplemental Table 2.

**Response:** We produce Supporting Table 2 to compliment the primers used to amplify the full lengths of the M and S segments. (Lines 131).

Reviewer #2: The manuscirpt requires to be thoroughly revised to reach the recquired clarity in data presentation

**Response:** We modified the results to reach the required clarity of data representation.

**Summary and General Comments**

Reviewer #1: This study aimed to analyze the phylogeography of hantaviruses detected in Rodent and Chiroptera. However, the data presented are insufficient to verify the authors' claims. While I expect the authors to be correct in their assertion, there is a disconnect between their results and conclusions, and I would like to see more careful analysis and support data that can adequately support their assertion.

**Response:** Phylogenetic trees can reflect the timeline of viral spread by showing the order of divergence between different strains. The article argues that it is possible to speculate on the phylogeographic consequences of viral spread in species based on this information.We've also revised some of the geographic extrapolations, so if there are any other conclusions that need updating, we'd appreciate it if you'd let us know.(Lines 239-244,284-287, 320-322)

In addition, numerous citations in the literature seem to employ papers that approximate but do not provide correct information. The authors' citations were partly correct and partly incorrect. Addressing such citations may hinder the smooth review process. The authors are urged to make good faith in the corrections.

For example, the first report of XSV was by Arai et al. and should be cited correctly. Gu et al. did not confirm or mention XSV in their paper.

**Response:** The issue regarding referencing errors has been resolved. We have removed duplicate descriptions of XSV origins from results and verified the other citations. (Lines 304-306).

Reviewer #2: The manuscript "Distribution and Genetic Characterization of Hantaviruses in Bats and Rodents from Yunnan" presents important and interesting findings, figures are clear and illustrative

However, the background data and discussion repeatedly refer to outdated references, text structure is difficult to follow and content is not clear, so, in spite of not having major request regarding the very topic and analyses, because of the extent of editorial revision needed I recommend "major revision"

**Response:** We will once again verify the accuracy of the citation, which reinforces the connection between the results and the conclusions. Because of the lack of reports of hantaviruses in Yunnan in recent years, we cited some earlier literature to describe the prevalence of hantaviruses in bats and rats in Yunnan. In addition, the reason for citing these Chinese journals was that we wanted to give a comprehensive picture of the prevalence of hantavirus in this region of Yunnan.

We have provided thorough and detailed responses to the experts' comments and suggestions. We would love to thank you for allowing us to resubmit a revised copy of themanuscript and we highly appreciate your time and consideration.

Thank you for your consideration. I look forward to hearing from you.

Sincerely,

Binghui Wang

No. 1168 Chunrong West Road, Yuhua Street, Chenggong District, Kunming City, Yunnan Province

Phone: 0086-13987140390

Email: [wangbh@kust.edu.cn](mailto:wangbh@kust.edu.cn)
