# Supplementary material for: Distribution and genetic characterization of hantaviruses in bats and rodents from Yunnan
Source: PLoS Negl Trop Dis. 2024 Aug 29;18(8):e0012437. doi: 10.1371/journal.pntd.0012437 (PMC11412632; doi:10.1371/journal.pntd.0012437)
Supplement: Attachment — Submitted filename: resubmission_cover_letter-6.26.docx [file pntd.0012437.s006.docx]

2024.6.26

Wen-Ping Guo

Academic Editor

*PLoS Neglected Tropical Diseases*

Dear Editor:

We wish to re-submit the manuscript titled “Distribution and Genetic Characterization of Hantaviruses in Bats and Rodents from Yunnan.” The manuscript ID is PNTD-D-23-01645R1.

We thank you and the reviewers for your thoughtful suggestions and insights. The manuscript has benefited from these insightful suggestions. I look forward to working with you and the reviewers to move this manuscript closer to publication in *PLoS Neglected Tropical Diseases*.

The manuscript has been rechecked and the necessary changes have been made in accordance with the reviewers’ suggestions.the modifications are highlighted in yellow in the revised manuscript. The main corrections in the manuscript and the responds to the reviewer’s comments are as follows:

**Response to Reviewer 's Comments**

**Methods**

Reviewer #1: (No Response)

Reviewer #2: The authors have accepted most of the suggestions and the revised manuscript is substantially improved, still, some comments remain:

lines 45, 46 - please, use the entire name prior to introducing acronyme (XSV, LSHV)

**Response:** We provide the full name of XSV and place the LSHV in the conclusion to present it.(line47).

line 51 - the correct name of the viral order in question is Bunyavirales, please, correct; considering the dynamic changing of the ICTV classification, it is already obsolete, so the authors might decide to delete it and keep just the viral family info.

Response: We have deleted “the Bunyaviralese”.(line 51-52).

line 53 - the segment is MEDIUM not MIDDLE

Response: We have changed it.(line 53)

line 65 - "increasing dyspnea on chest radiography" is not quite correct, dyspnea is a symptom, please, rephrase

Response: We have changed " increasing" with "worsening".(line 66)

**Results**

Reviewer #1: The LQV in Figure 3C is careless errors. LQV is presumed that the LSHV is presented in Figures 3A and 3B. I assume that this difference is one of the core elemental data of this study.The inclusion of careless errors in this most crucial aspect of the data significantly compromises the credibility of this study.

Response: We apologize for the error and have corrected the naming of LSHV in the Figure 3C.

Reviewer #2: lines 148-151 - positivity rates values in different specimens are confusing and not clearly prsented, please, rephrase

Response: We have harmonized the descriptions of the positivity rates in the Results.(line 146-152).

line 171 - Lineage 10 refers to SEOV, please, be precise in the title and through the text; also, the classification of SEOV into linegaes has not been explaned and elaborated nowhere in the manuscript, please, elaborate this either in the intorduction or briefly in the discussion section

Response: We will place the explanation of "lineage 10" after the results section and addressed the classification of Seoul virus in the introduction and Figrure 2 of our manuscript (line95-97,194-195).

**Conclusions**

Reviewer #1: In Figure 3, the authors present the amino acid homology of GP between strains of XSV as ranging from 92.72 to 94.24%. Additionally, the amino acid homology between LQUV and LSHV is presented as 91.89%. It is noteworthy that 92.72% homology of the sequences indicated the same virus, while 91.89% homology indicated the distinct from it. XSV has already exhibited a 7% difference in ICTV. It is evident that the criterion of a percentage difference does not apply in this case. The current situation does not preclude the possibility that the true host of LQUV exists separately and that spillover infections are being detected. It is acknowledged that the authors' judgment is valid; however, there are still some aspects that have not yet been clarified, and further discussion is necessary.

Response: Thank you for your insightful comments. We classify LSHV as a new species because its closest relative strain shows an amino acid homology greater than 7%. Currently, there are no reference strains in the database with a homology less than 7% to LSHV, whereas such instances exist among XSV strains. This distinction is a key factor in our classification approach.Additionally, our conclusion is primarily aimed at establishing LSHV as a novel species of Hantavirus. Discussing the possibility of spillover risks would require supported arguments, and we aim to ensure that our conclusions are well-grounded.

Reviewer #2: line 223 - still, blunt statements such as: "Hantavirus pulmonary syndrome, one of the most serious human diseases, has a significant impact on society" remained, please rephrase and specify in general, Conclusions are missing and are not clearly formulated

Response: Although the pathogenicity of hantavirus is mentioned in the introduction, the discussion of Hantavirus pulmonary syndrome seems unrelated to the subsequent topics covered in our paper. Therefore, we have removed that sentence and made adjustments to the sentence order to improve the coherence and focus of our discussion..

**Summary and General Comments**

Reviewer #1: The authors have responded to the review in a satisfactory manner, with the exception of a few instances where further clarification is required. I have identified specific areas where additional corrections are necessary. These are outlined in each part.

Reviewer #2: The manuscript "Distribution and Genetic Characterization of Hantaviruses in Bats and Rodents from Yunnan" presents important and interesting findings, figures are clear and illustrative

However, text structure is often difficult to follow and content is not clear, so, in spite of not having major request regarding the very topic and analyses, because of the extent of editorial revision needed I recommend "major revision"

**Response:** We have noted your concerns regarding the text structure and content clarity. In response to your recommendation for major revision, we have taken the following actions:

**1.Text Structure**: We have revised the structure of the titles in the results section to the following: ***Hantavirus Infections in Rodents and Bats; Genetic Characteristics of Hantavirus Strains; Identification of a Novel Rodent-Borne Seoul Virus Lineage; Phylogenetic Analysis of Full-Length M- and S-Segments of Bat-Borne Hantavirus***. (line156,179,198-199). This modification emphasizes the purpose of each result and clarifies the research content. Specifically, our results include:

**Hantavirus Infections in Rodents and Bats**: Detailed analysis of infection rates across different species and geographical locations, highlighting the prevalence of hantaviruses in various rodent and bat populations.

**Genetic Characteristics of Hantavirus Strains:** Comprehensive genetic analysis of the detected hantavirus strains, including sequence similarity and divergence among the strains.

**Identification of a Novel Rodent-Borne Seoul Virus Lineage:** Discovery and characterization of a new lineage of Seoul virus in rodents, providing insights into its unique genetic features and potential public health implications.

**Phylogenetic Analysis of Full-Length M- and S-Segments of Bat-Borne Hantavirus:** Phylogenetic relationships and evolutionary dynamics of bat-borne hantaviruses based on full-length M- and S-segments;Discovery and characterization of new geographic clusters of Xuan son virus and a novel hantavirus in bats

**2.Content Clarity**: We have removed redundant descriptions of trees in the results section to enhance clarity (lines 157-166, 180-184, 221-226). In the discussion section, we have eliminated parts with poor relevance and revised some descriptions to make the narrative more coherent (lines 228-243,305-307).

We believe these revisions address your concerns and significantly enhance the manuscript. We are committed to ensuring that the manuscript meets the journal's standards and are ready to make further adjustments if necessary. We would love to thank you for allowing us to resubmit a revised copy of themanuscript and we highly appreciate your time and consideration.

Thank you for your consideration. I look forward to hearing from you.

Sincerely,

Binghui Wang

No. 1168 Chunrong West Road, Yuhua Street, Chenggong District, Kunming City, Yunnan Province

Phone: 0086-13987140390

Email: [wangbh@kust.edu.cn](mailto:wangbh@kust.edu.cn)
