# Supplementary material for: Distribution and genetic characterization of hantaviruses in bats and rodents from Yunnan
Source: PLoS Negl Trop Dis. 2024 Aug 29;18(8):e0012437. doi: 10.1371/journal.pntd.0012437 (PMC11412632; doi:10.1371/journal.pntd.0012437)
Supplement: Attachment — Submitted filename: resubmission_cover_letter-7.24.docx [file pntd.0012437.s007.docx]

2024.7.26

Wen-Ping Guo

Academic Editor

*PLoS Neglected Tropical Diseases*

Dear Editor:

We wish to re-submit the manuscript titled “Distribution and Genetic Characterization of Hantaviruses in Bats and Rodents from Yunnan.” The manuscript ID is PNTD-D-23-01645R1.

We thank you and the reviewers for your thoughtful suggestions and insights. The manuscript has benefited from these insightful suggestions. I look forward to working with you and the reviewers to move this manuscript closer to publication in *PLoS Neglected Tropical Diseases*.

The manuscript has been rechecked and the necessary changes have been made in accordance with the reviewers’ suggestions.the modifications are highlighted in yellow in the revised manuscript. The main corrections in the manuscript and the responds to the reviewer’s comments are as follows:

**Response to Reviewer 's Comments**

line 94, bat-associated hantaviruses were also identified in Zhejiang and Hubei. (line 99-101)

lines 112-113, Chu xiong, Hong he, De hong, Nu jiang, Xi shuang ban na and Da li, should be Chuxiong, Honghe, Dehong, Nujiang, Xishuangbanna and Dali.(line 118-119)

line 115, add ℃ after -80. (line 121)

line120， Tianamp virus RNA kit, from which coporation?(line 126)

line 144, Rattus norvegicus, should be italicized(line 150-151)

line 151, add "and" before "the positive rate was 25%"(line 157)

line 155, "murinae", should be "Muridae"(line 161)

line 156, add "based on L gene sequences" before "was constructed"(line 162)

line 166, "RDRP sequences", should be L gene sequences(line 170)

line 164, add "other" before "two strains"(line 170)

line 167, add "Dode virus" before "in Pu'er"(line 173)

line 168, add "other" before "two sequenced strains". Which bat species were "the two sequenced strains" from?(line 174)

lines 166-175, "strains" should be replaced with "sequences"(line 172-181)

line 176, should be lineage, not lineages(line 182)

line 198, ull-length, mistake(line 205)

line 199, "hantaviruses (the ML trees produced similar topologies; Figs 3A and B). ",should be "hantaviruses. The ML trees produced similar topologies (Figs 3A and B)."(line 205-206)

lines 233 and 238, delete Seoul virus and Hantaan virus(line 240 and 245)

lines 239 and 240, the rodent species name should be italicized (line 246)

line 242, R. tanezumi, should be Apodemus agrarius(line 248)

line 265, delete "a lineage 5 variant or"(line 273)

lines 286-290, delete these sentences(line 296)

**Response:** The above changes have been marked in the manuscript, and the corresponding modifications have been made.

line 139, Poisson model, why was this model used?

**Response:** The reason we used the Poisson model is that the amino acid phylogenetic analysis serves as supplementary data to support the arguments in Figure 1. The Poisson model is more computationally efficient when dealing with smaller sample sizes, and its simplicity allows for faster and more intuitive calculations.(line 145)

line 183, how to define the lineage? In the M tree, lineage 4 did not form a distinct clade. ML trees based on partial S and M must be needed.

**Response:** Thank you for pointing this out. This is indeed an oversight in our analysis. We have revised the content, and the conclusion regarding the new lineage should be based on the combined analysis of the full-length M and S genes.(line 187-188)

line 197, 42 or 43? why XSVS from Pu'er were not selected to get the full S and M gene? add "related to" before 2 LQUVs

**Response:** This was our mistake; it has been corrected to 43 XSVs. The XSVs from Pu'er do not belong to a new lineage or a new geographical phylogroup, so there was no need to amplify the full-length genes. This has been clarified in the section "Genetic characteristics of Hantavirus strains." We have also added "related to."(line 203)

lines 211-214 should be revised

**Response:** We have revised the relevant statements to clarify that the new bat-borne hantaviruses exhibit 91.89% and 90.12% amino acid similarity to the full-length M and S genes of the most related strain, LQUV Longquan-Ra-90. This meets the ICTV classification criteria, thereby identifying it as a new hantavirus species.(line 216-222)

lines 220-223, which gens was compared with genus Loanvirus

**Response:** We have included an explanation that the amino acid differences in LSHV were determined in comparison to other bat-borne hantaviruses.(line 229)

lines 273-274, "The hosts of XSV are all of the genus Hipposideros." should be revised

**Response:** This statement is indeed too absolute. We only intended to indicate that the XSVs involved in the current study are all from the genus Hipposideros. To avoid any ambiguity, we have decided to delete this statement.

lines 275-278, "underwent a host-switching event with the local bat population", were bats within genus Hipposideros collected in this study positive for XSV?

**Response:** Most of the XSV-positive samples identified in this study were from H. gentilis, but we also found three samples from different species. Therefore, we hypothesize that a host-switching event may have occurred. This point has been clarified in the revised manuscript.(line 280-285)

line 279, batch? right?

**Response:** It seems that the use of "batch" was inappropriate in this context. We have replaced it with "the Nujiang bat samples from this study."(line 288-289)

line 286, imposible, why?

**Response:** Most of the XSV-positive samples identified in this study were from H. gentilis, but we also found three samples from different species. Therefore, we hypothesize that a host-switching event may have occurred. This point has been clarified in the revised manuscript.(line 293-296)

We believe these revisions address your concerns and significantly enhance the manuscript. We are committed to ensuring that the manuscript meets the journal's standards and are ready to make further adjustments if necessary. We would love to thank you for allowing us to resubmit a revised copy of themanuscript and we highly appreciate your time and consideration.

Thank you for your consideration. I look forward to hearing from you.

Sincerely,

Binghui Wang

No. 1168 Chunrong West Road, Yuhua Street, Chenggong District, Kunming City, Yunnan Province

Phone: 0086-13987140390

Email: [wangbh@kust.edu.cn](mailto:wangbh@kust.edu.cn)
