# Supplementary material for: Distribution and genetic characterization of hantaviruses in bats and rodents from Yunnan
Source: PLoS Negl Trop Dis. 2024 Aug 29;18(8):e0012437. doi: 10.1371/journal.pntd.0012437 (PMC11412632; doi:10.1371/journal.pntd.0012437)
Supplement: Attachment — Submitted filename: resubmission_cover_letter-7.31.docx [file pntd.0012437.s008.docx]

2024.7.31

Wen-Ping Guo

Academic Editor

*PLoS Neglected Tropical Diseases*

Dear Editor:

We wish to re-submit the manuscript titled “Distribution and Genetic Characterization of Hantaviruses in Bats and Rodents from Yunnan.” The manuscript ID is PNTD-D-23-01645R1.

We thank you and the reviewers for your thoughtful suggestions and insights. The manuscript has benefited from these insightful suggestions. I look forward to working with you and the reviewers to move this manuscript closer to publication in *PLoS Neglected Tropical Diseases*.

The manuscript has been rechecked and the necessary changes have been made in accordance with the reviewers’ suggestions.the modifications are highlighted in yellow in the revised manuscript. The main corrections in the manuscript and the responds to the reviewer’s comments are as follows:

**Response to Reviewer 's Comments**

1. the bat species name should be italicized, such as lines 277-278, please check carefully throughout the text

**Response:** Thank you for the reminder. We have revised the species names that were not in italics.（line 285-287，299）

2. lines 276-278 "Among the 43 XSV-positive samples in Nujiang, 40 were from H. gentilis, while the remaining 3 were from Rhinolophus sinicus (2 samples) and Hipposideros armiger (1 sample)." and 284 "Considering that its hosts include R. affinis", the bats name positive for XSV and LSHV should be also showed in result section.

**Response:** Thank you for the editor's reminder. In response to your suggestions, we have made the following two changes:

1. The abbreviations within the strain names already reflect the host species. For example, in Yunnan-Ra-015/China/2022, 'Ra' indicates that the host is Rhinolophus affinis. Since we submitted Hipposideros gentilis under the name Hipposideros pomona, we used 'Hp' in the strain names. However, throughout the manuscript, we consistently use Hipposideros gentilis for clarity.And we acknowledge the lack of clarity in our original description and have now added this information to the Results section. (line171-172,177-178)

Additionally, we have included the strain names in the discussion to provide clearer context regarding positive samples.(line 286-287,294).

2.We have revised Supplementary Tables 1 and 3 to make the positive cases clearer, ensuring a better correspondence between the species names and the strain names.

We believe these revisions address your concerns and significantly enhance the manuscript. We are committed to ensuring that the manuscript meets the journal's standards and are ready to make further adjustments if necessary. We would love to thank you for allowing us to resubmit a revised copy of themanuscript and we highly appreciate your time and consideration.

Thank you for your consideration. I look forward to hearing from you.

Sincerely,

Binghui Wang

No. 1168 Chunrong West Road, Yuhua Street, Chenggong District, Kunming City, Yunnan Province

Phone: 0086-13987140390

Email: [wangbh@kust.edu.cn](mailto:wangbh@kust.edu.cn)
