# Supplementary material for: Distribution and genetic characterization of hantaviruses in bats and rodents from Yunnan
Source: PLoS Negl Trop Dis. 2024 Aug 29;18(8):e0012437. doi: 10.1371/journal.pntd.0012437 (PMC11412632; doi:10.1371/journal.pntd.0012437)
Supplement: Peer review history

# Peer review: Decision Letter 0

**DOI:** 10.1371/journal.pntd.0012437.r001

**Authors:** Andrea Marzi, Wen-Ping Guo

4 Mar 2024

Dear Prof. Wang,

Thank you very much for submitting your manuscript "Distribution and Genetic Characterization of Hantaviruses in Bats and Rodents from Yunnan" for consideration at PLOS Neglected Tropical Diseases. As with all papers reviewed by the journal, your manuscript was reviewed by members of the editorial board and by several independent reviewers. In light of the reviews (below this email), we would like to invite the resubmission of a significantly-revised version that takes into account the reviewers' comments. 

We cannot make any decision about publication until we have seen the revised manuscript and your response to the reviewers' comments. Your revised manuscript is also likely to be sent to reviewers for further evaluation.

When you are ready to resubmit, please upload the following:

[1] A letter containing a detailed list of your responses to the review comments and a description of the changes you have made in the manuscript. Please note while forming your response, if your article is accepted, you may have the opportunity to make the peer review history publicly available. The record will include editor decision letters (with reviews) and your responses to reviewer comments. If eligible, we will contact you to opt in or out.

[2] Two versions of the revised manuscript: one with either highlights or tracked changes denoting where the text has been changed; the other a clean version (uploaded as the manuscript file). 

Important additional instructions are given below your reviewer comments.

Please prepare and submit your revised manuscript within 60 days. If you anticipate any delay, please let us know the expected resubmission date by replying to this email. Please note that revised manuscripts received after the 60-day due date may require evaluation and peer review similar to newly submitted manuscripts.

Thank you again for your submission. We hope that our editorial process has been constructive so far, and we welcome your feedback at any time. Please don't hesitate to contact us if you have any questions or comments.

Sincerely,

Wen-Ping Guo

Academic Editor

PLOS Neglected Tropical Diseases

Andrea Marzi

Section Editor

PLOS Neglected Tropical Diseases

***********************

Reviewer's Responses to Questions

**Key Review Criteria Required for Acceptance?**

As you describe the new analyses required for acceptance, please consider the following:

**Methods**

-Are the objectives of the study clearly articulated with a clear testable hypothesis stated?

-Is the study design appropriate to address the stated objectives?

-Is the population clearly described and appropriate for the hypothesis being tested?

-Is the sample size sufficient to ensure adequate power to address the hypothesis being tested?

-Were correct statistical analysis used to support conclusions?

-Are there concerns about ethical or regulatory requirements being met?

Reviewer #1: The authors have made phylogeographic claims. In such cases, I believe that geographical information such as map information is essential.

Although this study determined the full-length sequences of the S and M segments, it did not present any primer information used for this determination. If NGS is used, the method should be described; however, this method has not yet been described.

H. pomona, described by the authors, is now referred to by a different scientific name. Please check the reference and provide the correct species name.

Diversity 2021, 13, 218. https://doi.org/10.3390/d13050218

Reviewer #2: Objectives of the study are clearly articulated, study design is appropriate to address the stated objectives. Methods are clearly described and appropriate, additional clarify is needed regarding sampling. Sample size is adequate for this type of study. Phylogenetic methods are correct

Specific commenst:

Line 44 - please, refer to the current hantavirus taxonomy;

Line 50 - the statement "Hantaviruses originated from the black threaded gopher" seems incorect, please, revise and clarify ;

Line 68 - the statement "The last PUUV outbreak occurred in Germany in 2012" is not true, please, update the references and revise;

Line 69 - Dobrava virus (DOBV) - please, correct the full name to Dobrava-Belgrade virus

Line2 102,103 - the statement is unreferenced, please, clarify

In the Results sections analyses of fecal samples are presented yet no mention of this type of samples in the methods

--------------------

**Results**

-Does the analysis presented match the analysis plan?

-Are the results clearly and completely presented?

-Are the figures (Tables, Images) of sufficient quality for clarity?

Reviewer #1: The resolution of the figure and tables was poor and indiscernible (figures 1-3). Some action is needed.

The classification of LQUV and BRNV is mentioned, but it should be discussed using amino acid-based phylogenetic analysis, as well as DNA phylogenetic analysis. The authors may be correct in their assertion, but it is not clear whether they are misrepresenting the facts, and it is impossible to discern. This should be mentioned based on the phylogenetic analysis using amino acids.

In the phylogenetic analysis of hantaviruses in figure 1, there are several viruses that should be included as outgroups. Among them, Thottapalayam thottimvirus (TPMV), Nova mobatvirus (NVAV), Uluguru thottimvirus (ULUV), and Kilimanjaro thottimvirus (KMJV) must be included, and in this study, thottimvirus group was not added at all. In particular, ULUV and KMJV are the only viruses identified in Myosoricinae, so author must be included in the phylogenetic analysis of hantaviruses as a whole, but they were not included in this study.

In a normal phylogenetic analysis, multiple branches usually indicate inadequate analysis. There is a region of low bootslap (no numbers) and multifurcation downstream of bootstrap 86 in the authors' figure 1, Chiroptera region. Analysis of this portion of the region is entirely uncertain, and it is impossible to draw definitive conclusions from these results. Figure 1 also confirms the results of the low bootstrap values (no numbers) in areas where hantaviruses detected from each organism are heavily branched. This result alone could lead one to mistakenly believe that the large categories shown in figure 1 are not sufficiently reliable. Reanalysis of the authors' data with additional sequence data might provide results that support the authors' assertions.

Reviewer #2: Results are clearly presented, the figures are clear and of sufficient quality. Adjustnent regarding sampling is needed

Specific comments:

In the Method s section preparation of lung samples is described, yet Results are shown for fecal samples only? Please, apend and clarify

Two headings in the results section are consecutively numbered 3.3, please, correct

Lines 214, 215 "five strains from the 42 viruses in separate clusters and two strains with closer affinity to 215 LQUV in Nujiang were selected" - selected for what? please, append and clarify

--------------------

**Conclusions**

-Are the conclusions supported by the data presented?

-Are the limitations of analysis clearly described?

-Do the authors discuss how these data can be helpful to advance our understanding of the topic under study?

-Is public health relevance addressed?

Reviewer #1: Overall, the results were very interesting, but the phylogenetic and phylogeographic analyses were insufficient to fully understand the path to the conclusions. I request that the authors conduct the analysis again.

Reviewer #2: Conclusions are missing and are not clearly formulated

--------------------

**Editorial and Data Presentation Modifications?**

Use this section for editorial suggestions as well as relatively minor modifications of existing data that would enhance clarity. If the only modifications needed are minor and/or editorial, you may wish to recommend “Minor Revision” or “Accept”. 

Reviewer #1: As noted in the Methods section, primer information is not provided, but information on the deposited sequences is provided in supplemental Table 2.

Reviewer #2: The manuscirpt requires to be thoroughly revised to reach the recquired clarity in data presentation

--------------------

**Summary and General Comments**

Use this section to provide overall comments, discuss strengths/weaknesses of the study, novelty, significance, general execution and scholarship. You may also include additional comments for the author, including concerns about dual publication, research ethics, or publication ethics. If requesting major revision, please articulate the new experiments that are needed.

Reviewer #1: This study aimed to analyze the phylogeography of hantaviruses detected in Rodent and Chiroptera. However, the data presented are insufficient to verify the authors' claims. While I expect the authors to be correct in their assertion, there is a disconnect between their results and conclusions, and I would like to see more careful analysis and support data that can adequately support their assertion.

In addition, numerous citations in the literature seem to employ papers that approximate but do not provide correct information. The authors' citations were partly correct and partly incorrect. Addressing such citations may hinder the smooth review process. The authors are urged to make good faith in the corrections.

For example, the first report of XSV was by Arai et al. and should be cited correctly. Gu et al. did not confirm or mention XSV in their paper.

Reviewer #2: The manuscript "Distribution and Genetic Characterization of Hantaviruses in Bats and Rodents from Yunnan" presents important and interesting findings, figures are clear and illustrative

However, the background data and discussion repeatedly refer to outdated references, text structure is difficult to follow and content is not clear, so, in spite of not having major request regarding the very topic and analyses, because of the extent of editorial revision needed I recommend "major revision"

--------------------

PLOS authors have the option to publish the peer review history of their article (what does this mean?). If published, this will include your full peer review and any attached files.

If you choose “no”, your identity will remain anonymous but your review may still be made public.

**Do you want your identity to be public for this peer review?** For information about this choice, including consent withdrawal, please see our Privacy Policy.

Reviewer #1: No

Reviewer #2: Yes: Maja Stanojevic

Figure Files:

While revising your submission, please upload your figure files to the Preflight Analysis and Conversion Engine (PACE) digital diagnostic tool, https://pacev2.apexcovantage.com. PACE helps ensure that figures meet PLOS requirements. To use PACE, you must first register as a user. Then, login and navigate to the UPLOAD tab, where you will find detailed instructions on how to use the tool. If you encounter any issues or have any questions when using PACE, please email us at figures@plos.org.

Data Requirements:

Please note that, as a condition of publication, PLOS' data policy requires that you make available all data used to draw the conclusions outlined in your manuscript. Data must be deposited in an appropriate repository, included within the body of the manuscript, or uploaded as supporting information. This includes all numerical values that were used to generate graphs, histograms etc.. For an example see here: http://www.plosbiology.org/article/info%3Adoi%2F10.1371%2Fjournal.pbio.1001908#s5.

Reproducibility:

To enhance the reproducibility of your results, we recommend that you deposit your laboratory protocols in protocols.io, where a protocol can be assigned its own identifier (DOI) such that it can be cited independently in the future. Additionally, PLOS ONE offers an option to publish peer-reviewed clinical study protocols. Read more information on sharing protocols at https://plos.org/protocols?utm_medium=editorial-email&utm_source=authorletters&utm_campaign=protocols

# Author comment: Author response to Decision Letter 0

**DOI:** 10.1371/journal.pntd.0012437.r002

6 May 2024

Attachment Submitted filename: resubmission_cover_letter-5.3.docx

# Peer review: Decision Letter 1

**DOI:** 10.1371/journal.pntd.0012437.r003

**Authors:** Andrea Marzi, Wen-Ping Guo

30 May 2024

Dear Prof. Wang,

Thank you very much for submitting your manuscript "Distribution and Genetic Characterization of Hantaviruses in Bats and Rodents from Yunnan" for consideration at PLOS Neglected Tropical Diseases. As with all papers reviewed by the journal, your manuscript was reviewed by members of the editorial board and by several independent reviewers. In light of the reviews (below this email), we would like to invite the resubmission of a significantly-revised version that takes into account the reviewers' comments. 

We cannot make any decision about publication until we have seen the revised manuscript and your response to the reviewers' comments. Your revised manuscript is also likely to be sent to reviewers for further evaluation.

When you are ready to resubmit, please upload the following:

[1] A letter containing a detailed list of your responses to the review comments and a description of the changes you have made in the manuscript. Please note while forming your response, if your article is accepted, you may have the opportunity to make the peer review history publicly available. The record will include editor decision letters (with reviews) and your responses to reviewer comments. If eligible, we will contact you to opt in or out.

[2] Two versions of the revised manuscript: one with either highlights or tracked changes denoting where the text has been changed; the other a clean version (uploaded as the manuscript file). 

Important additional instructions are given below your reviewer comments.

Please prepare and submit your revised manuscript within 60 days. If you anticipate any delay, please let us know the expected resubmission date by replying to this email. Please note that revised manuscripts received after the 60-day due date may require evaluation and peer review similar to newly submitted manuscripts.

Thank you again for your submission. We hope that our editorial process has been constructive so far, and we welcome your feedback at any time. Please don't hesitate to contact us if you have any questions or comments.

Sincerely,

Wen-Ping Guo

Academic Editor

PLOS Neglected Tropical Diseases

Andrea Marzi

Section Editor

PLOS Neglected Tropical Diseases

***********************

Reviewer's Responses to Questions

**Key Review Criteria Required for Acceptance?**

As you describe the new analyses required for acceptance, please consider the following:

**Methods**

-Are the objectives of the study clearly articulated with a clear testable hypothesis stated?

-Is the study design appropriate to address the stated objectives?

-Is the population clearly described and appropriate for the hypothesis being tested?

-Is the sample size sufficient to ensure adequate power to address the hypothesis being tested?

-Were correct statistical analysis used to support conclusions?

-Are there concerns about ethical or regulatory requirements being met?

Reviewer #1: (No Response)

Reviewer #2: The authors have accepted most of the suggestions and the revised manuscript is substantially improved, still, some comments remain:

lines 45, 46 - please, use the netire mane prior to introducing acronyme (XSV, LSHV)

line 51 - the correct name of the viral order in question is Bunyavirales, please, correct; considering the dynamic changing of the ICTV classification, it is already obsolete, so the authors might decide to delete it and keep just the viral family info.

line 53 - the segment is MEDIUM not MIDDLE

line 65 - "increasing dyspnea on chest radiography" is not quite correct, dyspnea is a symptom, please, rephrase

--------------------

**Results**

-Does the analysis presented match the analysis plan?

-Are the results clearly and completely presented?

-Are the figures (Tables, Images) of sufficient quality for clarity?

Reviewer #1: The LQV in Figure 3C is careless errors. LQV is presumed that the LSHV is presented in Figures 3A and 3B. I assume that this difference is one of the core elemental data of this study.　The inclusion of careless errors in this most crucial aspect of the data significantly compromises the credibility of this study.

Reviewer #2: lines 148-151 - positivity rates values in different specimens are confusing and not clearly prsented, please, rephrase

line 171 - Lineage 10 refers to SEOV, please, be precise in the title and through the text; also, the classification of SEOV into linegaes has not been explaned and elaborated nowhere in the manuscript, please, elaborate this either in the intorduction or briefly in the discussion section

--------------------

**Conclusions**

-Are the conclusions supported by the data presented?

-Are the limitations of analysis clearly described?

-Do the authors discuss how these data can be helpful to advance our understanding of the topic under study?

-Is public health relevance addressed?

Reviewer #1: In Figure 3, the authors present the amino acid homology of GP between strains of XSV as ranging from 92.72 to 94.24%. Additionally, the amino acid homology between LQUV and LSHV is presented as 91.89%. It is noteworthy that 92.72% homology of the sequences indicated the same virus, while 91.89% homology indicated the distinct from it. XSV has already exhibited a 7% difference in ICTV. It is evident that the criterion of a percentage difference does not apply in this case. The current situation does not preclude the possibility that the true host of LQUV exists separately and that spillover infections are being detected. It is acknowledged that the authors' judgment is valid; however, there are still some aspects that have not yet been clarified, and further discussion is necessary.

Reviewer #2: line 223 - still, blunt statements such as: "Hantavirus pulmonary syndrome, one of the most serious human diseases, has a significant impact on society" remained, please rephrase and specify

in general, Conclusions are missing and are not clearly formulated

--------------------

**Editorial and Data Presentation Modifications?**

Use this section for editorial suggestions as well as relatively minor modifications of existing data that would enhance clarity. If the only modifications needed are minor and/or editorial, you may wish to recommend “Minor Revision” or “Accept”. 

Reviewer #1: (No Response)

Reviewer #2: The manuscirpt still requires revision, to reach the quality neede for PLOS Neglected Tropical Diseases

--------------------

**Summary and General Comments**

Use this section to provide overall comments, discuss strengths/weaknesses of the study, novelty, significance, general execution and scholarship. You may also include additional comments for the author, including concerns about dual publication, research ethics, or publication ethics. If requesting major revision, please articulate the new experiments that are needed.

Reviewer #1: The authors have responded to the review in a satisfactory manner, with the exception of a few instances where further clarification is required. I have identified specific areas where additional corrections are necessary. These are outlined in each part.

Reviewer #2: The manuscript "Distribution and Genetic Characterization of Hantaviruses in Bats and Rodents from Yunnan" presents important and interesting findings, figures are clear and illustrative

However, text structure is often difficult to follow and content is not clear, so, in spite of not having major request regarding the very topic and analyses, because of the extent of editorial revision needed I recommend "major revision"

--------------------

PLOS authors have the option to publish the peer review history of their article (what does this mean?). If published, this will include your full peer review and any attached files.

If you choose “no”, your identity will remain anonymous but your review may still be made public.

**Do you want your identity to be public for this peer review?** For information about this choice, including consent withdrawal, please see our Privacy Policy.

Reviewer #1: No

Reviewer #2: Yes: Maja Stanojevic

Figure Files:

While revising your submission, please upload your figure files to the Preflight Analysis and Conversion Engine (PACE) digital diagnostic tool, https://pacev2.apexcovantage.com. PACE helps ensure that figures meet PLOS requirements. To use PACE, you must first register as a user. Then, login and navigate to the UPLOAD tab, where you will find detailed instructions on how to use the tool. If you encounter any issues or have any questions when using PACE, please email us at figures@plos.org.

Data Requirements:

Please note that, as a condition of publication, PLOS' data policy requires that you make available all data used to draw the conclusions outlined in your manuscript. Data must be deposited in an appropriate repository, included within the body of the manuscript, or uploaded as supporting information. This includes all numerical values that were used to generate graphs, histograms etc.. For an example see here: http://www.plosbiology.org/article/info%3Adoi%2F10.1371%2Fjournal.pbio.1001908#s5.

Reproducibility:

To enhance the reproducibility of your results, we recommend that you deposit your laboratory protocols in protocols.io, where a protocol can be assigned its own identifier (DOI) such that it can be cited independently in the future. Additionally, PLOS ONE offers an option to publish peer-reviewed clinical study protocols. Read more information on sharing protocols at https://plos.org/protocols?utm_medium=editorial-email&utm_source=authorletters&utm_campaign=protocols

# Author comment: Author response to Decision Letter 1

**DOI:** 10.1371/journal.pntd.0012437.r004

27 Jun 2024

Attachment Submitted filename: resubmission_cover_letter-6.26.docx

# Editor report: Decision Letter 2

**DOI:** 10.1371/journal.pntd.0012437.r005

**Authors:** Andrea Marzi, Wen-Ping Guo

22 Jul 2024

Dear Prof. Wang,

Thank you very much for submitting your manuscript "Distribution and Genetic Characterization of Hantaviruses in Bats and Rodents from Yunnan" for consideration at PLOS Neglected Tropical Diseases. As with all papers reviewed by the journal, your manuscript was reviewed by members of the editorial board and by several independent reviewers. In light of the reviews (below this email), we would like to invite the resubmission of a significantly-revised version that takes into account the reviewers' comments. 

line 94, bat-associated hantaviruses were also identified in Zhejiang and Hubei

lines 112-113, Chu xiong, Hong he, De hong, Nu jiang, Xi shuang ban na and Da li, should be Chuxiong, Honghe, Dehong, Nujiang, Xishuangbanna and Dali

line 115, add ℃ after -80

line120， Tianamp virus RNA kit, from which coporation?

line 139, Poisson model, why was this model used?

line 144, Rattus norvegicus, should be italicized

line 151, add "and" before "the positive rate was 25%"

line 155, "murinae", should be "Muridae"

line 156, add "based on L gene sequences" before "was constructed"

line 166, "RDRP sequences", should be L gene sequences

line 164, add "other" before "two strains"

line 167, add "Dode virus" before "in Pu'er"

line 168, add "other" before "two sequenced strains". Which bat species were "the two sequenced strains" from?

lines 166-175, "strains" should be replaced with "sequences"

line 176, should be lineage, not lineages

line 183, how to define the lineage? In the M tree, lineage 4 did not form a distinct clade. ML trees based on partial S and M must be needed. 

line 197, 42 or 43? why XSVS from Pu'er were not selected to get the full S and M gene? add "related to" before 2 LQUVs

line 198, ull-length, mistake

line 199, "hantaviruses (the ML trees produced similar topologies; Figs 3A and B). ",should be "hantaviruses. The ML trees produced similar topologies (Figs 3A and B)."

lines 211-214 should be revised

lines 220-223, which gens was compared with genus Loanvirus

lines 233 and 238, delete Seoul virus and Hantaan virus

lines 239 and 240, the rodent species name should be italicized

line 242, R. tanezumi, should be Apodemus agrarius

line 265, delete "a lineage 5 variant or"

lines 273-274, "The hosts of XSV are all of the genus Hipposideros." should be revised

lines 275-278, "underwent a host-switching event with the local bat population", were bats within genus Hipposideros collected in this study positive for XSV? 

line 279, batch? right?

line 286, imposible, why?

lines 286-290, delete these sentences

We cannot make any decision about publication until we have seen the revised manuscript and your response to the reviewers' comments. Your revised manuscript is also likely to be sent to reviewers for further evaluation.

When you are ready to resubmit, please upload the following:

[1] A letter containing a detailed list of your responses to the review comments and a description of the changes you have made in the manuscript. Please note while forming your response, if your article is accepted, you may have the opportunity to make the peer review history publicly available. The record will include editor decision letters (with reviews) and your responses to reviewer comments. If eligible, we will contact you to opt in or out.

[2] Two versions of the revised manuscript: one with either highlights or tracked changes denoting where the text has been changed; the other a clean version (uploaded as the manuscript file). 

Important additional instructions are given below your reviewer comments.

Please prepare and submit your revised manuscript within 60 days. If you anticipate any delay, please let us know the expected resubmission date by replying to this email. Please note that revised manuscripts received after the 60-day due date may require evaluation and peer review similar to newly submitted manuscripts.

Thank you again for your submission. We hope that our editorial process has been constructive so far, and we welcome your feedback at any time. Please don't hesitate to contact us if you have any questions or comments.

Sincerely,

Wen-Ping Guo

Academic Editor

PLOS Neglected Tropical Diseases

Andrea Marzi

Section Editor

PLOS Neglected Tropical Diseases

***********************

line 94, bat-associated hantaviruses were also identified in Zhejiang and Hubei

lines 112-113, Chu xiong, Hong he, De hong, Nu jiang, Xi shuang ban na and Da li, should be Chuxiong, Honghe, Dehong, Nujiang, Xishuangbanna and Dali

line 115, add ℃ after -80

line120， Tianamp virus RNA kit, from which coporation?

line 139, Poisson model, why was this model used?

line 144, Rattus norvegicus, should be italicized

line 151, add "and" before "the positive rate was 25%"

line 155, "murinae", should be "Muridae"

line 156, add "based on L gene sequences" before "was constructed"

line 166, "RDRP sequences", should be L gene sequences

line 164, add "other" before "two strains"

line 167, add "Dode virus" before "in Pu'er"

line 168, add "other" before "two sequenced strains". Which bat species were "the two sequenced strains" from?

lines 166-175, "strains" should be replaced with "sequences"

line 176, should be lineage, not lineages

line 183, how to define the lineage? In the M tree, lineage 4 did not form a distinct clade. ML trees based on partial S and M must be needed. 

line 197, 42 or 43? why XSVS from Pu'er were not selected to get the full S and M gene? add "related to" before 2 LQUVs

line 198, ull-length, mistake

line 199, "hantaviruses (the ML trees produced similar topologies; Figs 3A and B). ",should be "hantaviruses. The ML trees produced similar topologies (Figs 3A and B)."

lines 211-214 should be revised

lines 220-223, which gens was compared with genus Loanvirus

lines 233 and 238, delete Seoul virus and Hantaan virus

lines 239 and 240, the rodent species name should be italicized

line 242, R. tanezumi, should be Apodemus agrarius

line 265, delete "a lineage 5 variant or"

lines 273-274, "The hosts of XSV are all of the genus Hipposideros." should be revised

lines 275-278, "underwent a host-switching event with the local bat population", were bats within genus Hipposideros collected in this study positive for XSV? 

line 279, batch? right?

line 286, imposible, why?

lines 286-290, delete these sentences

Figure Files:

While revising your submission, please upload your figure files to the Preflight Analysis and Conversion Engine (PACE) digital diagnostic tool, https://pacev2.apexcovantage.com. PACE helps ensure that figures meet PLOS requirements. To use PACE, you must first register as a user. Then, login and navigate to the UPLOAD tab, where you will find detailed instructions on how to use the tool. If you encounter any issues or have any questions when using PACE, please email us at figures@plos.org.

Data Requirements:

Please note that, as a condition of publication, PLOS' data policy requires that you make available all data used to draw the conclusions outlined in your manuscript. Data must be deposited in an appropriate repository, included within the body of the manuscript, or uploaded as supporting information. This includes all numerical values that were used to generate graphs, histograms etc.. For an example see here: http://www.plosbiology.org/article/info%3Adoi%2F10.1371%2Fjournal.pbio.1001908#s5.

Reproducibility:

To enhance the reproducibility of your results, we recommend that you deposit your laboratory protocols in protocols.io, where a protocol can be assigned its own identifier (DOI) such that it can be cited independently in the future. Additionally, PLOS ONE offers an option to publish peer-reviewed clinical study protocols. Read more information on sharing protocols at https://plos.org/protocols?utm_medium=editorial-email&utm_source=authorletters&utm_campaign=protocols

# Author comment: Author response to Decision Letter 2

**DOI:** 10.1371/journal.pntd.0012437.r006

26 Jul 2024

Attachment Submitted filename: resubmission_cover_letter-7.24.docx

# Editor report: Decision Letter 3

**DOI:** 10.1371/journal.pntd.0012437.r007

**Authors:** Andrea Marzi, Wen-Ping Guo

30 Jul 2024

Dear Prof. Wang,

Thank you very much for submitting your manuscript "Distribution and Genetic Characterization of Hantaviruses in Bats and Rodents from Yunnan" for consideration at PLOS Neglected Tropical Diseases. As with all papers reviewed by the journal, your manuscript was reviewed by members of the editorial board and by several independent reviewers. The reviewers appreciated the attention to an important topic. Based on the reviews, we are likely to accept this manuscript for publication, providing that you modify the manuscript according to the review recommendations. 

1. the bat species name should be italicized, such as lines 277-278, please check carefully throughout the text

2. lines 276-278 "Among the 43 XSV-positive samples in Nujiang, 40 were from H. gentilis, while the remaining 3 were from Rhinolophus sinicus (2 samples) and Hipposideros armiger (1 sample)." and 284 "Considering that its hosts include R. affinis", the bats name positive for XSV and LSHV should be also showed in result section.

Please prepare and submit your revised manuscript within 30 days. If you anticipate any delay, please let us know the expected resubmission date by replying to this email. 

When you are ready to resubmit, please upload the following:

[1] A letter containing a detailed list of your responses to all review comments, and a description of the changes you have made in the manuscript. 

Please note while forming your response, if your article is accepted, you may have the opportunity to make the peer review history publicly available. The record will include editor decision letters (with reviews) and your responses to reviewer comments. If eligible, we will contact you to opt in or out

[2] Two versions of the revised manuscript: one with either highlights or tracked changes denoting where the text has been changed; the other a clean version (uploaded as the manuscript file).

Important additional instructions are given below your reviewer comments. 

Thank you again for your submission to our journal. We hope that our editorial process has been constructive so far, and we welcome your feedback at any time. Please don't hesitate to contact us if you have any questions or comments.

Sincerely,

Wen-Ping Guo

Academic Editor

PLOS Neglected Tropical Diseases

Andrea Marzi

Section Editor

PLOS Neglected Tropical Diseases

***********************

1. the bat species name should be italicized, such as lines 277-278, please check carefully throughout the text

2. lines 276-278 "Among the 43 XSV-positive samples in Nujiang, 40 were from H. gentilis, while the remaining 3 were from Rhinolophus sinicus (2 samples) and Hipposideros armiger (1 sample)." and 284 "Considering that its hosts include R. affinis", the bats name positive for XSV and LSHV should be also showed in result section.

Figure Files:

While revising your submission, please upload your figure files to the Preflight Analysis and Conversion Engine (PACE) digital diagnostic tool, https://pacev2.apexcovantage.com. PACE helps ensure that figures meet PLOS requirements. To use PACE, you must first register as a user. Then, login and navigate to the UPLOAD tab, where you will find detailed instructions on how to use the tool. If you encounter any issues or have any questions when using PACE, please email us at figures@plos.org.

Data Requirements:

Please note that, as a condition of publication, PLOS' data policy requires that you make available all data used to draw the conclusions outlined in your manuscript. Data must be deposited in an appropriate repository, included within the body of the manuscript, or uploaded as supporting information. This includes all numerical values that were used to generate graphs, histograms etc.. For an example see here: http://www.plosbiology.org/article/info%3Adoi%2F10.1371%2Fjournal.pbio.1001908#s5.

Reproducibility:

To enhance the reproducibility of your results, we recommend that you deposit your laboratory protocols in protocols.io, where a protocol can be assigned its own identifier (DOI) such that it can be cited independently in the future. Additionally, PLOS ONE offers an option to publish peer-reviewed clinical study protocols. Read more information on sharing protocols at https://plos.org/protocols?utm_medium=editorial-email&utm_source=authorletters&utm_campaign=protocols

References

Please review your reference list to ensure that it is complete and correct. If you have cited papers that have been retracted, please include the rationale for doing so in the manuscript text, or remove these references and replace them with relevant current references. Any changes to the reference list should be mentioned in the rebuttal letter that accompanies your revised manuscript. If you need to cite a retracted article, indicate the article's retracted status in the References list and also include a citation and full reference for the retraction notice.

# Author comment: Author response to Decision Letter 3

**DOI:** 10.1371/journal.pntd.0012437.r008

2 Aug 2024

Attachment Submitted filename: resubmission_cover_letter-7.31.docx

# Editor report: Decision Letter 4

**DOI:** 10.1371/journal.pntd.0012437.r009

**Authors:** Andrea Marzi, Wen-Ping Guo

5 Aug 2024

Dear Prof. Wang,

We are pleased to inform you that your manuscript 'Distribution and Genetic Characterization of Hantaviruses in Bats and Rodents from Yunnan' has been provisionally accepted for publication in PLOS Neglected Tropical Diseases.

Before your manuscript can be formally accepted you will need to complete some formatting changes, which you will receive in a follow up email. A member of our team will be in touch with a set of requests.

Please note that your manuscript will not be scheduled for publication until you have made the required changes, so a swift response is appreciated.

IMPORTANT: The editorial review process is now complete. PLOS will only permit corrections to spelling, formatting or significant scientific errors from this point onwards. Requests for major changes, or any which affect the scientific understanding of your work, will cause delays to the publication date of your manuscript.

Should you, your institution's press office or the journal office choose to press release your paper, you will automatically be opted out of early publication. We ask that you notify us now if you or your institution is planning to press release the article. All press must be co-ordinated with PLOS.

Thank you again for supporting Open Access publishing; we are looking forward to publishing your work in PLOS Neglected Tropical Diseases.

Best regards,

Wen-Ping Guo

Academic Editor

PLOS Neglected Tropical Diseases

Andrea Marzi

Section Editor

PLOS Neglected Tropical Diseases

***********************************************************

# Editor report: Acceptance letter

**DOI:** 10.1371/journal.pntd.0012437.r010

**Authors:** Andrea Marzi, Wen-Ping Guo

21 Aug 2024

Dear Prof. Wang,

We are delighted to inform you that your manuscript, "Distribution and Genetic Characterization of Hantaviruses in Bats and Rodents from Yunnan," has been formally accepted for publication in PLOS Neglected Tropical Diseases.

We have now passed your article onto the PLOS Production Department who will complete the rest of the publication process. All authors will receive a confirmation email upon publication.

The corresponding author will soon be receiving a typeset proof for review, to ensure errors have not been introduced during production. Please review the PDF proof of your manuscript carefully, as this is the last chance to correct any scientific or type-setting errors. Please note that major changes, or those which affect the scientific understanding of the work, will likely cause delays to the publication date of your manuscript. Note: Proofs for Front Matter articles (Editorial, Viewpoint, Symposium, Review, etc...) are generated on a different schedule and may not be made available as quickly.

Soon after your final files are uploaded, the early version of your manuscript will be published online unless you opted out of this process. The date of the early version will be your article's publication date. The final article will be published to the same URL, and all versions of the paper will be accessible to readers.

Thank you again for supporting open-access publishing; we are looking forward to publishing your work in PLOS Neglected Tropical Diseases. 

Best regards,

Shaden Kamhawi

co-Editor-in-Chief

PLOS Neglected Tropical Diseases

Paul Brindley

co-Editor-in-Chief

PLOS Neglected Tropical Diseases
